# Olfactory systems across mosquito species

**DOI:** 10.1007/s00441-020-03407-2

**Published:** 2021-01-21

**Authors:** Matthew Wheelwright, Catherine R. Whittle, Olena Riabinina

**Affiliations:** Department of Biosciences, Durham University, Stockton Road, Durham, DH1 3LE UK

**Keywords:** Receptors, Neurons, Olfactory organs, Brain, Evolution, Sensory ecology, Neuroethology, Mosquitoes

## Abstract

There are 3559 species of mosquitoes in the world (Harbach 2018) but, so far, only a handful of them have been a focus of olfactory neuroscience and neurobiology research. Here we discuss mosquito olfactory anatomy and function and connect these to mosquito ecology. We highlight the least well-known and thus most interesting aspects of mosquito olfactory systems and discuss promising future directions. We hope this review will encourage the insect neuroscience community to work more broadly across mosquito species instead of focusing narrowly on the main disease vectors.

## Introduction

Pick up any paper about mosquitoes and the first sentence of it will tell you about the diseases and deaths that mosquitoes bring to humans. It is no wonder then that most of the mosquito research so far has focussed on the species that transmit diseases, such as *Anopheles gambiae*, *Aedes aegypti*, *Culex quinquefasciatus* and a handful of other common disease vectors. But this is only a part of the story—out of 3500+ mosquito species (Harbach 2018) separated by up to 145–200 million years of evolution (Chen et al. 2015; Hao et al. 2017) most do not transmit diseases and many are not interested in humans (e.g., Reeves et al. 2018) or do not blood-feed altogether (Miyagi 1981; O’Meara 1985; Rattanarithikul et al. 2007). The part of their olfactory repertoire devoted to host-seeking is likely to differ between these species (Wolff and Riffell 2018). Another part, dedicated to finding flowers for nectar feeding, will be adapted to recognise the volatiles from the flowering plants available in the species’ habitat (Nikbakhtzadeh et al. 2014; Zeng et al. 2019; Peach et al. 2019; Lahondère et al. 2019; Dekel et al. 2019a). The factors that determine an ideal oviposition site are also different—the absence of predator hydrocarbons for *Culiseta longiareolata* (Silberbush et al. 2010), bacterial volatiles for *Aedes aegypti* (Ponnusamy et al. 2008) and a leaf of the host pitcher plant for *Wyeomyia smithii* (Heard 1994). The most overlooked aspect of mosquito ecology—pheromones—is now receiving more attention (Mozūraitis et al. 2020) and some of these pheromones are likely to be strongly species-specific. Finally, larval mosquitoes also employ olfaction to recognise a variety of odorants (Xia et al. 2008; Sun et al. 2020a) the ecological relevance of which is still largely unknown.

Studying the olfactory systems of mosquitoes allows us to develop specific smell-based traps and repellents for those species that transmit deadly diseases. Equally, mosquitoes present us with unique opportunities to study unexplored processes that regulate olfactory sensitivity, rapid evolution of olfactory receptors and olfactory signals (Neafsey et al. 2015), relations between olfaction and speciation (Coetzee et al. 2013; Bradshaw et al. 2018) and the effect of climate change, urbanisation and invasions on sensory systems (Balkew et al. 2020; Rose et al. 2020). We may wish to look beyond the olfactory receptors and study the processing of signals in the mosquito brain, which opens up a whole new set of questions concerning multisensory integrations and feedbacks (Vinauger et al. 2019), the importance of trade-offs between brain areas devoted to different sensory modalities (Keesey et al. 2019) and the effects of learning (Lutz et al. 2017; Vinauger et al. 2018; Wolff et al. 2019).

In this review, we do not aim to provide a comprehensive summary of mosquito olfactory research to date. Instead, we aim to highlight the least explored and most fascinating facts and phenomena that we believe will define the future research on mosquito olfaction. We are, of course, biased.

## Olfactory organs

Olfactory perception in mosquitoes and other insects starts from their peripheral olfactory organs. These are traditionally defined by the expression of olfactory receptors (see the “Olfactory receptors” section below), neuronal responses and contribution to behavioural responses to airborne odorants. In adult mosquitoes, olfactory receptor expression has been localised to the antennae, maxillary palps and proboscis in *Anopheles* (Pitts et al. 2004, 2011; Athrey et al. 2017; Saveer et al. 2018), *Aedes* (Melo et al. 2004; Bohbot et al. 2007; Sparks et al. 2014; Matthews et al. 2016; Lombardo et al. 2017), *Toxorhynchites* (Zhou et al. 2014 (only antenna and maxillary palps were studied)) and *Culex* (Xia and Zwiebel 2006; Leal et al. 2013), implying that all three of these are peripheral olfactory organs. Electrophysiological and behavioural experiments support this conclusion (e.g., Kwon et al. 2006; Ghaninia et al. 2008; Saveer et al. 2018).

**Fig. 1:**
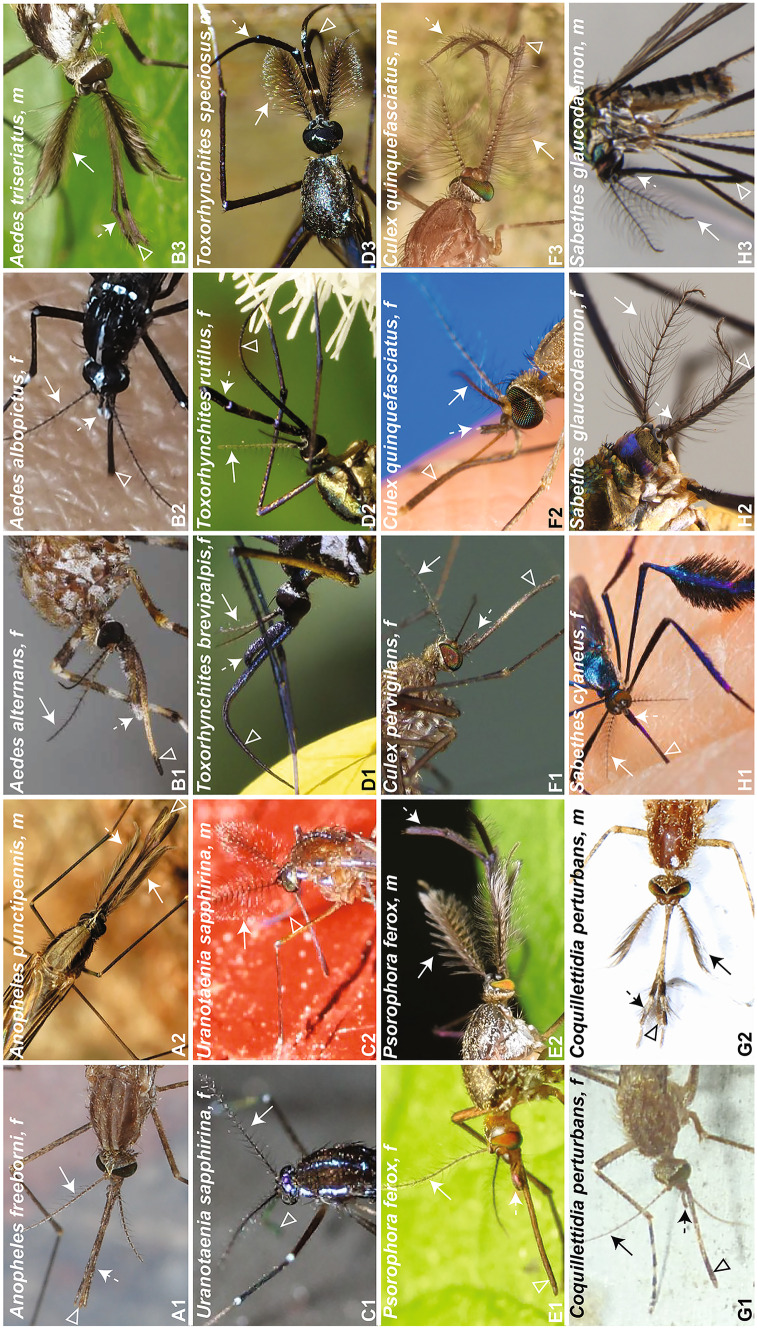
Olfactory organs of mosquitoes. Solid arrows point to antennae, dashed arrows point to maxillary palps, arrowheads point to proboscises. f—female, m—male. Image credits: A1. *Anopheles freeborni* female (https://www.inaturalist.org/observations/171373, Don Loarie), A2. *Anopheles punctipennis* male (https://www.inaturalist.org/observations/48198941, Katja Schulz), B1. *Aedes alternans* female (https://www.inaturalist.org/observations/50594863, Wendy Moore), B2. *Aedes albopictus* female (https://www.inaturalist.org/observations/32721364, Zygy), B3. *Aedes triseratus* male (https://www.inaturalist.org/observations/44504824, skitterbug); C1. *Uranotaenia sapphirina* female (https://www.inaturalist.org/observations/50204034, Arturo Santos), C2. *Uranotaenia sapphirina* male (https://www.inaturalist.org/observations/24801804, Even Dankowicz), D1. *Toxorhynchites brevipalpis* female (https://www.inaturalist.org/observations/45377361, Alan Manson), D2. *Toxorhynchites rutilus* female (https://www.inaturalist.org/observations/21372632, Katja Schulz), D3. *Toxorhynchites speciosus* male (https://www.inaturalist.org/observations/18770512, Steve Kerr); E1. *Psorophora* *ferox* female (https://www.inaturalist.org/observations/20379672, Katja Schulz), E2. *Psorophora ferox* male (https://www.inaturalist.org/observations/9620348, skitterbug), F1. *Culex pervigilans* female (https://www.inaturalist.org/observations/54584455, Steve Kerr), F2. *Culex quinquefasciatus* female (https://phil.cdc.gov/Details.aspx?pid=4734, James Gathany), F3. *Culex quinquefasciatus* male (https://www.inaturalist.org/observations/23073942, skitterbug); G1. *Coquillettidia perturbans* female (https://www.inaturalist.org/observations/19518594, Even Dankowicz), G2. *Coquillettidia perturbans* male (https://www.inaturalist.org/observations/6867156, Judy Gallagher), H1. *Sabethes cyaneus* female (https://phil.cdc.gov/Details.aspx?pid=20514, James Gathany), H2. *Sabethes glaucodaemon* female, and H3. *Sabethes glaucodaemon* male (both Image library of Coleção de Culicidae-Fundação Oswaldo Cruz, Rio de Janeiro, Brazil (CCULI))

The overall morphology of adult mosquito antennae and maxillary palps is similar across species, often with pronounced sexual dimorphisms. For example, males of *Aedes*, *Anopheles*, *Coquillettidia*, *Culex*, *Psorophora* and *Toxorhynchites* species have large bushy antennae (Fig. 1, A1-G2) whereas females of the same species have more slender antennae. Interestingly, antennal sexual dimorphisms are minimal in some species, such as *Sabethes* mosquitoes (Fig. 1, H1-H3). Apart from having an olfactory function, antennae also mediate sound detection in mosquitoes (e.g., Warren et al. 2010; Lapshin 2012; Windmill et al. 2018; Su et al. 2018) and the evolution of overall antennal morphology has most likely been driven by demands of both olfaction and hearing.

The overall morphology of maxillary palps differs across species and between sexes. For example, male *Anopheles* have long club-shaped maxillary palps, whereas females have cylindrical palps that are slightly shorter than those of males (Fig 1, A1-A2). *Sabethes*’ palps are very short and similar in males and females (Fig. 1, H1-H3) In *Aedes*, *Culex*, *Toxorhynchites* and *Psorophora* males sport elaborate maxillary palps that curve upwards, whereas females have straight maxillary palps that are much shorter than the proboscis (Fig 1, B1-B3 and D1-F3). Maxillary palps carry chemosensory sensilla and mechanosensory bristles and are thus, like antennae, both olfactory and mechanosensory organs (McIver 1971; McIver and Hudson 1972). However, mechanosensitivity is accomplished at the level of individual sensilla and not the entire organ, as is the case for antennae. The role of different maxillary palp morphologies thus remains unclear.

The morphology of the proboscis presents the most interesting case of the three olfactory organs and is related to the role of the proboscis in blood intake. The mosquito proboscis usually consists of 6 organs (a pair of maxillae with teeth-like structures, a pair of mandibles, a needle-like labrum and a hypopharynx) encased in a labium that ends in a labellum (see e.g., Fig. 1 in Choo et al. 2015). In females of blood-feeding species, all 6 organs are about the same length as the labium and they enter the skin during blood-feeding. The length of the maxillae and mandibles varies in males of these species (Wahid et al. 2003), and stylet innervation is strongly sexually dimorphic in *Aedes aegypti* (Jové et al. 2020). The males of blood-feeding species are not able to pierce skin and do not blood-feed. In fact, blood significantly reduced the survival of male *Culex quinquefasciatus* in a laboratory experiment (Nikbakhtzadeh et al. 2016). Interestingly, both males and females of non-bloodfeeding *Malaya* and *Topomyia* species have completely lost both mandibles and maxillae (Wahid et al. 2003). The proboscises of some non-bloodfeeding species have strikingly different morphologies compared with blood-feeding mosquitoes. For example, *Malaya* mosquitoes have an unusual proboscis, enlarged at its distal end (Rattanarithikul et al. 2007). These mosquitoes feed on bamboo juice that they obtain via trophallaxis from *Crematogaster* ants (Miyagi 1981). *Toxorhynchites* mosquitoes also do not feed on blood and have a very distinct long curved proboscis (Fig. 1, D1-D3) presumably to facilitate nectar feeding from plants.

The appearance of the labium is similar between males and females of blood-feeding species (Fig. 1). In addition, in *Anopheles gambiae* females, the proboscis is connected by a dense cuticle to the posterior surface of the head capsule, whereas in males it is not connected (OR, personal observation). This anatomical feature presumably helps to exert the necessary force to insert the stylet into and extract it from, the skin. Labella of *Anopheles gambiae* harbour equal numbers of olfactory sensory neurons (OSNs) in males and females (Riabinina et al. 2016).

The antennae, maxillary palps and proboscis are not functionally equivalent substrates for their olfactory neurons. The differences lie in the patterns of brain innervation (see “Structure and function of the antennal lobe and subesophageal zone” below) and in the specific groupings of olfactory neurons within their sensilla (see “Inhibition in the peripheral olfactory system: mechanisms and ecological relevance” below). In addition, morphologically different types of olfactory sensilla cover the three organs (e.g., McIver 1971; McIver and Hudson 1972; Khalifa et al. 2013), although the importance of sensillar morphology remains unclear.

Mosquito larvae also exhibit chemosensory behaviours and express olfactory receptors (Bohbot et al. 2007; Xia et al. 2008; Liu et al. 2010; Scialò et al. 2012; Bui et al. 2019; Melo et al. 2020; Lutz et al. 2020; Sun et al. 2020a). *Anopheles gambiae* harbour olfactory neurons in their antennae and maxillary palps (Xia et al. 2008; Riabinina et al. 2016; Sun et al. 2020a). In contrast to adult mosquitoes, in the larvae the number of neurons and the overall anatomy of antennae and maxillary palps are not sexually dimorphic in this species (O.R., personal observation). Indeed, the ecological needs of male and female larvae of *Anopheles gambiae* are likely to be identical. It would be very interesting to explore the anatomy of the larval olfactory systems in mosquito species that do not blood-feed. In these species, the nutrient accumulation needed for egg production is shifted to the larval stage and thus, female larvae have potentially different needs to the male ones.

There have been no reports of olfactory behaviours in mosquito pupae that are, like larvae, aquatic and mobile but in contrast to larvae do not feed.

## Olfactory receptors

### Types of olfactory receptors and chemosensory proteins in mosquitoes

Olfactory receptor genes have been identified and annotated, to a varying extent, in the genomes of 2 *Aedes*, 19 *Anopheles* and 1 *Culex* species (https://vectorbase.org/) but this list is growing continuously. Additional information about mosquito receptors comes from gene expression studies, e.g., in *Toxorhynchites amboinensis* (Zhou et al. 2014) and *Anopheles coluzzii* (Athrey et al. 2017). Comparative analysis of *Anopheles* genomes found that chemoreceptor genes in these species are among the fastest evolving (Neafsey et al. 2015). At the same time, the odorant receptor-coreceptor ORCO is very highly conserved in insects (Missbach et al. 2014) and is the only chemoreceptor with a known 3D structure (Butterwick et al. 2018).

The three main classes of receptors involved in mosquito olfaction, similarly to other insects, are gustatory receptors (GRs), ionotropic receptors (IRs) and odorant receptors (ORs) (e.g., Leal 2013; Suh et al. 2014). IRs and ORs are sensitive to a range of odorants (e.g., Xia et al. 2008; Carey et al. 2010), whereas the role of GRs in olfaction is limited to the detection of CO_2_ and other volatile odorants via a heterotrimeric complex made up of G1, G2 and G3 in *Aedes* sp. and *Culex* sp. and G22, G23 and G24 in *Anopheles* sp. (Hill et al. 2002; Robertson and Kent 2009; Tauxe et al. 2013; Younger et al. 2020). Transient receptor potential channels (TRPs), sensory neuron membrane proteins (SNMPs) and pickpocket (ppk) ion channels are also expressed in the chemosensory organs of *Aedes aegypti* (Bohbot et al. 2014; Tallon et al. 2019), *Culex quinquefasciatus* (Leal et al. 2013; Taparia et al. 2017) and *Anopheles gambiae* (Zelle et al. 2013), although their role in mosquito olfactory processing is not yet established. Odorant binding proteins (OBPs), chemosensory proteins (CSPs) and CheA/B proteins are involved in olfactory processing in other insects and may also play an olfactory roles in mosquitoes (Ben-Shahar et al. 2010; Leal 2013; Bohbot et al. 2014).

### Receptor expression and co-expression: novel findings

ORCO expression patterns have been studied in adult and larval *Anopheles gambiae* (Xia et al. 2008; Riabinina et al. 2016) and *Aedes aegypti* (Melo et al. 2004; Degennaro et al. 2013; Won Jung et al. 2015) by employing immunohistochemistry*, in situ* hybridisation and genetic tools. The same methods were used to establish expression patterns of GR4 and IR7a in *Aedes aegypti* (Jové et al. 2020). These works established expression of odorant receptors in the antennae, maxillary palps and proboscis of mosquitoes, in line with electrophysiological recordings (Kwon et al. 2006; Qiu et al. 2006; Lu et al. 2007). However, it remained unclear whether ORs, IRs and GRs are expressed in separate groups of neurons or if they may be co-expressed in the same cell. A large body of work in *Drosophila* argues for separate expression patterns (e.g., Benton et al. 2009), with very few exceptions (Dobritsa et al. 2003; Goldman et al. 2005; Couto et al. 2005; Fishilevich et al. 2005). This conclusion has been challenged by recent intriguing findings in *Drosophila* that IR and OR receptors are very broadly co-expressed and occur in the same neurons (Task et al. 2020). This is also true for *Aedes aegypti* mosquitoes (Younger et al. 2020), which has extensive implications relating to the processing of naturally occurring odorant mixtures, such as human body odor, which activate both OR (McBride et al. 2014) and IR receptors (Pitts et al. 2017).

A drastic difference in the expression of chemosensory proteins can be seen between larvae and adult mosquitoes. Possibly because of their reduced suite of olfactory-based behaviours, larvae show a smaller repertoire of expressed olfactory receptors. For example, whereas the adults of *Anopheles gambiae* and *Aedes aegypti* express 79 and 80 ORs respectively (Hill et al. 2002; Ruel et al. 2019), their larvae have a much smaller repertoire with *Anopheles gambiae* expressing 12 ORs (4 of which are larval specific) and at least 4 IRs (Xia et al. 2008; Liu et al. 2010) and *Aedes aegypti* expressing 23 ORs (15 of which are larval specific) (Bohbot et al. 2007) in their antennae. ORCO is also expressed in the maxillary palps of *Anopheles gambiae* larvae (Riabinina et al. 2016) but expression of other ORs has not been studied in this chemosensory organ.

A recent paper on *Anopheles gambiae* described an interesting feature of mosquito ORs, which is not shared by *Drosophila* (Maguire et al. 2020). This study focused on AgOR2, a receptor that is sensitive to indole, phenols, benzaldehyde and other aromatic compounds and is highly conserved between mosquito species (Carey et al. 2010; Bohbot et al. 2011). When AgOR2 was ectopically expressed in ORCO-positive olfactory sensory neurons where their native ORs were already present, the original ORs (but not ORCO) were downregulated and the ectopic OR2 was also not functional, leading to a total loss of odorant sensitivity in the ORCO neurons (Maguire et al. 2020). This finding suggests a fine-tuned mechanism that regulates expression levels of ORs in *Anopheles gambiae* and is disrupted by unnaturally high levels of OR2 protein. While in *Anopheles gambiae* OR2 overexpression or ORCO mutation does not lead to dramatic reduction of the size of the antennal lobe (Maguire et al. 2020; Sun et al. 2020b), ORCO mutation affects antennal lobe size in clonal and ponerine ants (Trible et al. 2017; Yan et al. 2017; Ryba et al. 2020) and, to a lesser extent, in the moth *Manduca sexta* (Fandino et al. 2019)*.* It therefore appears that there is a continuous spectrum of *ORCO*–*OR function*–*ORN development* interactions amongst insects, despite high conservation of ORCO. In mammals, OR expression is also crucial for the development of olfactory neurons and allows for correct mapping to the corresponding regions of the olfactory bulb (e.g., Lodovichi and Belluscio 2012).

Even more intriguing is that, unlike in *Drosophila* (with a few notable exceptions (Vosshall and Stocker 2007)), the olfactory neurons of some mosquitoes naturally co-express several odorant receptors. For instance, one neuronal type in the blunt trichoid sensilla of *Anopheles gambiae* co-expresses AgOR13, AgOR15, AgOR17 and AgOR55 (Schultze et al. 2014; Karner et al. 2015) and around half of these neurons also express AgOR16 and AgOR47, which means that some neurons can express up to six different olfactory receptors in the same cell (Karner et al. 2015). It is possible that the receptor interaction feedback system is only active after a receptor has been translated (Maguire et al. 2020). Since the 6 naturally co-expressed ORs form a gene cluster (Karner et al. 2015), they share the same regulatory elements and are all transcribed onto the same polycistronic mRNA (Karner et al. 2015), thus avoiding downregulation by the feedback system (Maguire et al. 2020). The prevalence of OR gene clusters in the genomes of mosquitoes could therefore be essential for functional expression of ORs (Fox et al. 2002; Hill et al. 2002).

### Enantio-selectivity

Some mosquito ORs show enantio-selectivity, which means that they have different affinities to the two forms, R and S, of a chiral molecule (Bohbot and Dickens 2009; Cook et al. 2011; Dekel et al. 2016; Huff and Pitts, 2019). For instance, cells expressing AgOR29 in *Anopheles gambiae* show a larger response to a mixture of (R)-(−)- and (S)-( +)-linalool compared with (R)-(−)-linalool alone. This suggests that AgOR29 is more sensitive to (S)-(+)-linalool than to (R)-(−)-linalool (Huff and Pitts 2019). In contrast, AaOR8 of *Aedes aegypti* is more sensitive to (R)-1-octen-3-ol than to (S)-1-octen-3-ol (Bohbot and Dickens 2009), which translates to a larger neuronal response to (R)-1-octen-3-ol than to (S)-1-octen-3-ol as well as a behavioural preference (Cook et al. 2011). This increased sensitivity to (R)-1-octen-3-ol is shared by *Culex quinquefasciatus* (Cook et al. 2011). In contrast to *Aedes aegypti*, *Culex quinquefasciatus* shows a higher aversion to (R)-1-octen-3-ol (Cook et al. 2011). The difference in the behavioural valence of 1-octen-3-ol may be due to the fact that it is an important host cue for mosquitoes. Interestingly, cattle produce 80–92% of (R)-1-octen-3-ol and 8–20% of (S)-1-octenol (Hall et al. 1984). Mosquitoes that feed on mammalian hosts, such *Aedes aegypti*, may thus have evolved attraction to it, whereas species that feed on avian hosts, such as *Culex quinquefasciatus*, are repelled by it (Cook et al. 2011). However, the OR8 of *Toxorhynchites amboinensis* is also enantio-selective and shows increased sensitivity to (R)-1-octen-3-ol (Dekel et al. 2016) despite the fact that this species does not feed on blood (Collins and Blackwell 2000). (R)-1-octen-3-ol could potentially be produced more than (S)-1-octen-3-ol by other sources of 1-octen-3-ol in the environment, such as flowering plants (Knudsen et al. 1993; Syed and Guerin 2004). For example, one plant species, *Lantana camara*, produces a mixture consisting of 95% (R)-1-octen-3-ol and 5% (S)-1-octenol (Syed 2002; Syed and Guerin 2004). While we do not know whether 1-octen-3-ol is produced by plants on which *Toxorhynchites* sp. feeds, there have only been a few observations of *Toxorhynchites* sp. feeding as adults (Collins and Blackwell 2000). It is possible that floral compounds consisting of more (R)-1-octen-3-ol than (S)-1-octenol could lead to the enantio-selectivity of OR8. It would be interesting to explore enantio-selectivity and its ecological significance for other mosquito ORs.

### Chemosensory proteins: OBPs and CheA/B

The ability for a mosquito to detect an odorant depends not only on the receptors expressed in the olfactory sensory neurons but also on accessory proteins in the perireceptor environment. In a sensillum, dendrites of olfactory sensory neurons are surrounded by an aqueous solution known as the sensillar lymph (Ishida et al. 2002). Hydrophobic odorants are insoluble in the lymph and thus need to be transported by another molecule to the receptor binding sites. This is done by odorant binding proteins (OBPs) (Leal 2013).

The role of OBPs was investigated in *Culex quinquefasciatus* where it was found that CquiOBP1 RNAi knock-down reduced the EAG responses to the mosquito oviposition pheromone (MOP) and indole by half (Pelletier et al. 2010). Similarly, knockdown of AgambOBP1 in *Anopheles gambiae* (Biessmann et al. 2010) and Obp37/39 in *Aedes albopictus* (Deng et al. 2013) abolished EAG responses to indole. OBP genes and transcripts have been also identified in *Aedes aegypti* (Zhou et al. 2008; Sengul and Tu 2010), *Anopheles sinensis* (He et al. 2016), *Anopheles stephensi* (Sengul and Tu 2008), *Anopheles quadriannulatus* (Sengul and Tu 2008), *Anopheles arabiensis* (Li et al. 2005), *Anopheles culicifacies* (Das et al. 2018) and *Anopheles funestus* (Xu et al. 2010), often expressed outside of the chemosensory organs per se. While there is no doubt that OBPs can bind odorants (a property that has been utilised for chemical detector devices (e.g. Cali and Persaud 2020), the role of OBPs in the insect olfactory system is less well understood. For example, *Drosophila* ab8 sensilla that normally express Obp28a retained or even increased their sensitivity to odorants after Obp28a knock-out (Larter et al. 2016). This result was supported by a subsequent study in *Drosophila* that found that basiconic sensilla showed no reduction in response to odorants when all their native OBPs were not expressed (Xiao et al. 2019). Pairing between OBPs/OBPs and OBPS/ORs is another unresolved puzzle that has been studied in *Anopheles gambiae* (Schultze et al. 2013) and *Drosophila melanogaster* (summarised in Xiao et al. 2019). OBPs may be expressed alone or co-expressed in various combinations of up to 4 OBPs that do not have a one-to-one correlation with OR expression patterns.

It appears that not all OBPs are the same: some of them have an essential function in the olfactory system, some have a modulatory function and some—no function. The structure of the sensilla themselves may provide one of the answers to this puzzle, as sensillar pore tubules in some sensilla dramatically reduce the travel path of an odorant through the lymph and thus the role of OBPs in these sensilla (Larter et al. 2016). Another possibility may lie in the structure of OBPs: currently, OBPs are separated into Classic, Minus-C, Plus-C and Atypical/two-domain categories based on the number of cysteine residues they contain (Manoharan et al. 2013). Indeed, apart from capturing odorant molecules, OBPs are also involved in odorant release, development regeneration, and other processes (Pelosi et al. 2018).

Another group of accessory proteins that are potentially involved in chemosensation are CheA and CheB proteins. These proteins were mainly studied in *Drosophila* but 3 members of this gene family are expressed in the antennae and maxillary palps of *Aedes albopictus* (AALF012531, AALF021726 and Ae2-204624_FR6_8–206) (Lombardo et al. 2017) and 13 (10 CheA and 3 CheB proteins) are expressed in the maxillary palps of *Aedes aegypti* (Bohbot et al. 2014). These proteins are involved in the perception of cuticular hydrocarbons during courtship in *Drosophila* (Park et al. 2006). However, there is evidence for (Park et al. 2006) and against (Lin et al. 2005; Ben-Shahar et al. 2010) the role of these proteins in fly mating behaviour. While the molecular function of Che A/B proteins is not known definitively (Lombardo et al. 2017), they could interact with DEG/EnaC channels to modulate their responses (Ben-Shahar et al. 2010).

### Evolution of olfactory receptors

The expression and rapid evolution of chemosensory proteins in mosquitoes often parallels the ecological relevance of chemicals that the proteins bind. For example, several studies have focused on the differences between anthropophilic and zoophilic *Aedes* mosquitoes (McBride et al. 2014). *Aedes aegypti aegypti* feeds on humans, while its forest-dwelling counterpart *Aedes aegypti formosus* is zoophilic (McBride et al. 2014) and inhabits forest areas of Africa, sometimes close to the human dwellings where *Aedes aegypti aegypti* is found (Rose et al. 2020). The two species may be found sympatrically in man-made habitats (Futami et al. 2020). AeOR4 receptor detects sulcatone, the prevalent component of human odour and is upregulated in the *Aedes aegypti aegypti* compared with *Aedes aegypti formosus* (McBride et al. 2014). The genetic differences between the two subspecies are focused in the region of chromosome 1 where the AeOR4 gene locus is located (Rose et al. 2020). Similarly, populations of *Aedes albopictus,* which have been moved to novel areas through human activity, show upregulation of OR100 and OR47-N2 compared with predominantly zoophilic populations from its native range (Gomulski et al. 2020). *Aedes aegypti* homologs of these receptors are upregulated in anthropophilic *Aedes aegypti aegypti*, suggesting that increased sensitivity to human odors may play a role in adaptations to new environments. It would be intriguing to investigate whether the recent spread of Asian *Anopheles stephensi* to the Horn of Africa (Seyfarth et al. 2019; Balkew et al. 2020) has been accompanied by a similar upregulation of ORs and IRs sensitive to human-derived volatiles.

To examine the olfactory repertoires of species that display a wider diversity of life history traits, recent studies compared mosquito species that feed on blood and those that do not, such as *Toxorhynchites amboinensis* (Zhou et al. 2014; Dekel et al. 2016, 2019b, a). These studies identified ORs that are most highly conserved among species: OR2 and OR10. These receptors detect indole and skatole, compounds derived from animal hosts, plants for nectar feeding and suitable oviposition sites (Dekel et al. 2019b). They are also important odorants for larvae since *Aedes aegypti* larvae express OR2 in their antennae along with another supersensitive, larval-specific, skatole receptor, AeOR9 (Ruel et al. 2019). Surprisingly, *Toxorhynchites amboinensis* expresses a receptor for sulcatone (TambOR4) despite not feeding on blood (Dekel et al. 2019a), which highlights the possibility that sulcatone is potentially used for behaviours other than host-seeking (Dekel et al. 2019a). Further studies could not only provide a better idea of the ecological relevance of many odorants but could also provide an insight into the evolutionary history of mosquito olfactory systems by identifying other odorant receptors that are highly conserved among species.

## Neurons and brain

### Inhibition in the peripheral olfactory system: mechanisms and ecological relevance

Responses of olfactory receptor neurons in *Drosophila* (e.g., Wilson 2013) and mosquitoes (e.g., Qiu et al. 2006; Ghaninia et al. 2007) are defined by the receptors they express. Neurons that occupy the same sensillum also modulate each other’s responses—a phenomenon known as lateral, or ephaptic, inhibition (Su et al. 2012). In the rare cases when two different functional odorant receptors co-exist in the same neuron, the responses of the neuron to an odorant may depend on both receptors. We name this phenomenon “intraneuron interaction” (Xu et al. 2019). Additionally, the nature of chemical ligands that bind to a receptor affects the receptor’s conductivity and its response to ligands—we call this “intrareceptor interaction” (Xu et al. 2019).

The process of lateral inhibition was initially investigated in *Drosophila*, looking at the ab3 sensillum (Su et al. 2012). In one experiment, a background odorant, methyl hexanoate, was supplied to stimulate sustained firing of one neuron (ab3A), whilst an odor pulse of 2-heptanone was simultaneously supplied to a neighbouring neuron (ab3B). Addition of the superimposed odor pulse substantially reduced the recorded response from ab3A in a dose-dependent manner. However, inhibition was not observed when ab3B was genetically ablated, ruling out direct inhibition at the receptor level. Repeating this procedure using capitate peg sensilla in *Anopheles gambiae* maxillary palps, with CO_2_ as the background odorant and 1-octen-3-ol superimposed, yielded the same effect (Su et al. 2012). Supporting results were found through testing a diverse range of *Drosophila* sensilla types (large basiconic, small basiconic, coeloconic and trichoid sensilla) with up to four ORNs each (Su et al. 2012). Furthermore, different species with compartmentalised receptor neurons, such as beetle sensilla, have demonstrated inhibitory effects (Nikonov and Leal 2002). These findings taken together suggest that lateral inhibition is a robust and broadly observed phenomenon among insects and plays an important role in the olfactory system of various mosquito species.

There remains debate over the mechanism for lateral inhibition; however, it is thought unlikely that synapses or gap junctions are involved; ephaptic transmission, also known as ephaptic coupling, seems to offer a more plausible explanation (Su et al. 2012; Xu et al. 2019; Pannunzi and Nowotny 2020). The extracellular electrical potential of a sensillum’s microenvironment (the sensillar lymph) is affected by ion flows, particularly the movement of cations into the neuron during depolarisation. Changes to this extracellular electric field influence the membrane potential and therefore the occurrence of action potentials of neurons within the same sensillum, meaning a neuron is suppressed through hyperpolarisation if a co-located neuron is activated (van der Goes van Naters 2013). Research in *Drosophila* (Zhang et al. 2019) has expanded on the proposed mechanism, suggesting an asymmetry in lateral inhibition, whereby the dominant ORN in a sensillum, that being the bigger neuron with characteristically larger spike amplitudes, exerts greater ephaptic effects and is less sensitive to them from other neurons. As such, the less dominant neuron has a reduced likelihood of reaching the action potential threshold when the more dominant ORN is stimulated (Zhang et al. 2019). It is generally observed across insect species that dominant neurons detect attractive odorants and non-dominant neurons detect aversive odorants (Ng et al. 2020). Sensilla are also thought to be organised antagonistically such that their ORNs detect stimuli from ecologically related sources that require opposing behavioural responses; for example ab4A and ab4B neurons in *Drosophila* promote and inhibit egg laying respectively (Ng et al. 2020).

At a more peripheral level there is evidence, though limited, that intrareceptor and intraneuron inhibition modulate odor responses (Xu et al. 2019). One receptor in *Culex quinquefasciatus*, CquiOR32, generates excitatory (inward) currents when activated by a range of ligands, such as cyclohexanone, methyl salicylate and 2-methyl-2-thiazoline. However, inhibitory (outward/reverse) currents are also elicited in this same receptor in a dose-dependent manner by a range of odorants including artificial insect repellents DEET and IR3535, as well as fenchone and eucalyptol (Xu et al. 2019). The authors reason that the chemical dissimilarity between agonists and inhibitors means it is unlikely that both bind to the same orthosteric receptor site, so suggest the involvement of an allosteric modulator (Xu et al. 2019). The exact mechanism remains unknown, though it is possible that inhibitory ligands cause the closing of certain channels that would usually be open to allow an anion influx and depolarisation. Alternatively, they may cause an influx of cations, with Cl^-^ a proposed candidate for involvement (Xu et al. 2019).

Intraneuron inhibition occurs when multiple receptors in the same neuron interact with each other. Experiments with transgenic *Drosophila* that ectopically expressed *Culex* receptor CquiOR32 in all ORCO+ neurons demonstrated that eucalyptol-induced inhibitory currents through CquiOR32 cancelled out excitatory methyl salicylate-induced currents through DmelOR98a (Xu et al. 2019). Similar effects were observed during SSR recordings in *Culex quinquefasciatus* and *Aedes aegypti* SST2 sensilla in response to excitatory cyclohexanone and inhibitory eucalyptol (Xu et al. 2019). It is worth noting that this type of inhibition relies on a single neuron expressing multiple different receptors, a phenomenon subject to current debate but thought to occur very rarely in flies and more frequently in mosquitoes (see **Receptor expression and co-expression: novel findings**) (Karner et al. 2015).

The inhibitory mechanisms described here have a range of possible advantages, particularly concerning mixture processing. Mixtures have an emergent perceptual quality, meaning that an ORN’s response to a mixture cannot necessarily be predicted by summing the ORN’s responses to that mixture’s individual components. Inhibitory effects enhance the dynamic range of ORN responses to aid in accurately determining concentration ratios of odors of mixtures (Pannunzi and Nowotny 2020). Another challenge of mixture perception is distinguishing between odors from a single source and odors that emanate from different sources (Pannunzi and Nowotny 2020). An odor plume is composed of filaments such that odorants from co-localised sources are found in the same filaments, whilst those from separate sources reach the receptors in different filaments. Therefore, only odors from a single source are sufficiently spatially and temporally synchronised to cause depolarisation of their corresponding ORNs sufficiently close together for ephaptic effects to exert an influence (Murlis et al. 1992). Furthermore, the unique temporal dynamics of ORN response spikes allow mixtures of compounds that promote excitatory and inhibitory responses to be distinguished from a single excitatory stimulus of lower intensity, regardless of stimulus duration or concentration (Su et al. 2011). Inhibitory effects may also aid in suppressing sustained but irrelevant, odors to focus on the most relevant stimuli, even if they are transient (Ng et al. 2020). Similarly, they are thought to allow particular inputs to be efficiently filtered out at the peripheral level (Zhang et al. 2019; Ng et al. 2020) with dominant ORNs being selectively favoured, potentially increasing neuronal sensitivity to specific, prioritised chemicals. The importance of valence is highlighted by antagonistic neuronal organisation and it is possible that in some cases, ephaptic inhibition serves to enhance behavioural attraction and suppress repulsion (Ng et al. 2020). In *Drosophila*, inhibitory mechanisms have been studied at the AL level (Silbering and Galizia 2007) but to date almost all mosquito studies have focused on peripheral mechanisms. Recently, however, a study using *Aedes aegypti* demonstrated symmetric lateral inhibition between two glomeruli, LC2 and AM2, which respond to the attractive orchid scent nonanal and the neutral orchid scent lilac aldehyde respectively (Lahondère et al. 2019). Inhibitory mechanisms in higher brain areas would be an interesting topic for future research (Pannunzi and Nowotny 2020).

### Structure and function of the antennal lobe and subesophageal zone

**Fig. 2:**
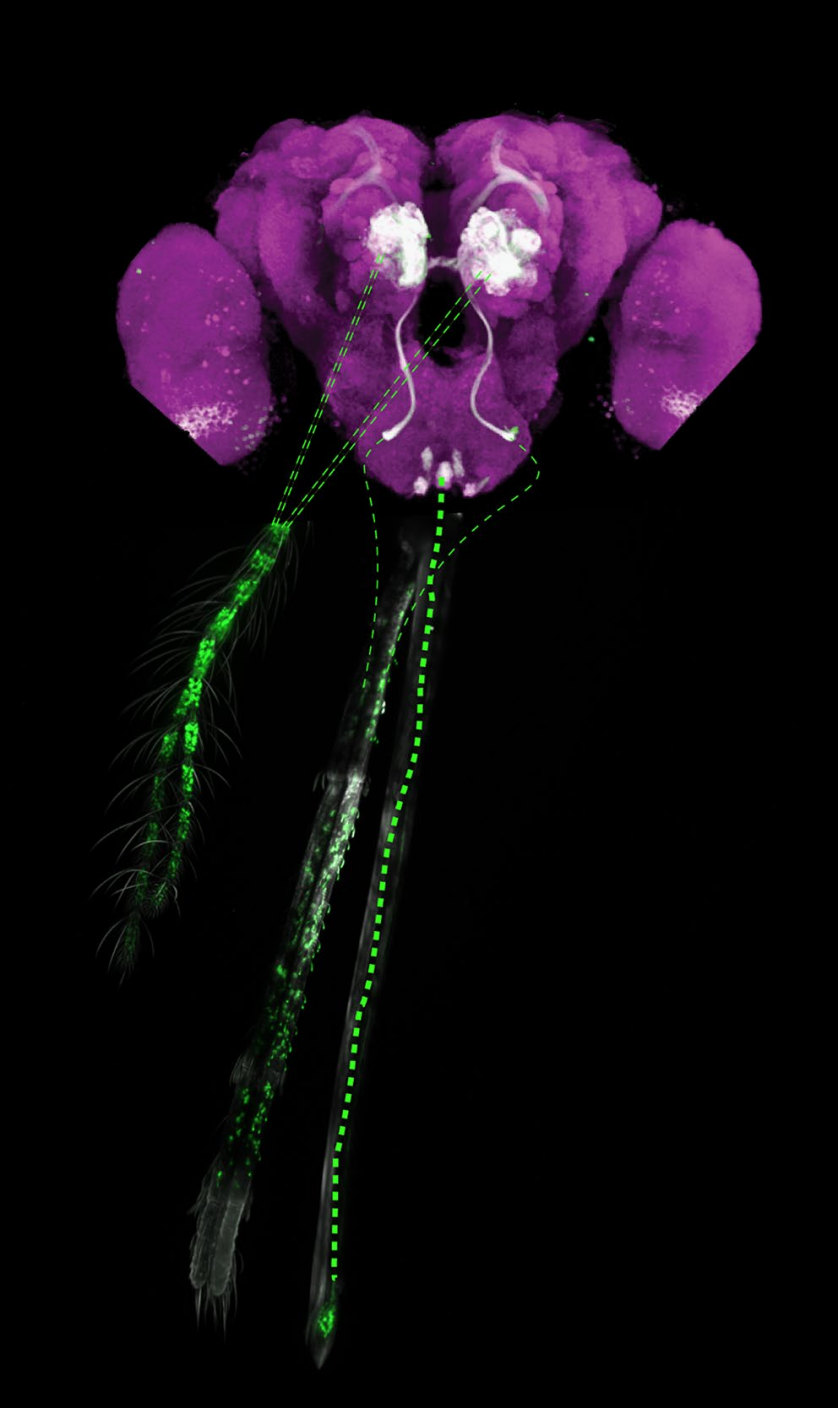
Olfactory innervations of *Anopheles gambiae* brain. Axons of olfactory receptor neurons located in the antennae and maxillary palps (thin green dashed lines) innervate specific subsets of glomeruli in the antennal lobe (AL). Axons of olfactory neurons located in the labellum (thick green dotted line) innervate 8 glomeruli-like structures in the suboesophageal zone (SEZ). The image depicts the brain and peripheral olfactory organs of an adult female *Anopheles gambiae.* Cell bodies of ORCO+ neurons are labelled with GFP

The antennal lobe (AL), studied most extensively in *Drosophila*, acts as an olfactory “relay system”, receiving input from olfactory sensory neurons (OSNs) then sending the information to higher brain centres via projection neurons (PNs) (Jefferis et al. 2007). Within the AL, local neurons (LNs) form a connective network between glomeruli. Glomeruli represent functional pieces of olfactory information, which combine to encode a spatial odor map for translation to and then interpretation by higher brain areas. The AL of *Anopheles gambiae* and *Aedes aegypti* is innervated by olfactory neurons with cell bodies in the antenna and maxillary palps (Fig. 2) (Riabinina et al. 2016; Raji et al. 2019; Lahondère et al. 2019). In *Aedes aegypti*, ORCO+ neurons from the stylet also project to the AL (Won Jung et al. 2015). Around half of ORNs innervating the AL in *Anopheles gambiae* are ORCO+, whilst the remainder are speculated to express IRs (Riabinina et al. 2016). Projection neurons have not been studied in mosquitoes but in *Drosophila* they target the mushroom body (MB) and the lateral horn (LH)—brain areas that most likely retained their olfactory processing role in mosquitoes.

In *Drosophila*, all ORNs of the same class target a single glomerulus for innervation, thereby producing a mapping system where one glomerulus corresponds with one OR, if a one receptor-one neuron principle is accepted (Komiyama and Luo 2006). In *Anopheles gambiae*, the number of expressed OR genes is notably greater than the number of ORCO+ glomeruli and therefore ORN classes, assuming that each glomerulus is innervated by a single class of ORNs (Riabinina et al. 2016). This observation has three plausible explanations: it may be that ORNs that express different ORs converge on the same glomerulus, or that some ORs are not expressed in olfactory neurons and thus have an alternative function outside of their usual role in olfactory processing, or it may imply some degree of OR co-expression in the same neuron. These explanations are not mutually exclusive.

Such a seemingly disorderly system, where glomeruli have inputs stemming from multiple ORN classes/receptors, may have arisen for several reasons. In some cases, gene duplication could have resulted in co-expressed ORs that respond to very similar odorants (Karner et al. 2015). This could potentially provide increased sensitivity to an odor, in effect amplifying its signal. Alternatively, a one-to-one mapping system may not be required to discriminate between each component of an ecologically relevant mixture. Instead, a neuron may have several receptors that together are broadly tuned to a blend of odors and work collectively to elicit a behavioural response (Karner et al. 2015).

ORNs of *Anopheles gambiae* also innervate the subesophageal zone (SEZ) (Fig. 2) (Riabinina et al. 2016), the brain region associated in *Drosophila* with gustatory input and feeding behaviours (Kendroud et al. 2018). Specifically, in *Anopheles gambiae* ORs from the labella on the proboscis innervate eight SEZ glomeruli. Of these, six appeared strongly and two appeared weakly labelled when investigated using anti-GFP antibody staining (Riabinina et al. 2016). Much of the remaining SEZ area is likely innervated by gustatory and mechanosensory neurons (Ignell et al. 2005). It is well documented that gustatory neurons project to the SEZ via the pharyngeal and labial nerves (Ignell and Hansson 2005) whilst neurons expressing the ppk301 receptor for freshwater detection and blood sensors IR7a/f also project to the SEZ, in this case from the proboscis and pharynx (Matthews et al. 2019; Jové et al. 2020). Based on these findings, it is hypothesised that the SEZ may act as a centre for the integration of olfactory and gustatory information, particularly during blood-feeding (see the “Multisensory integration and host-seeking” section).

The overall structure of the larval brain closely resembles that of the adults, with clearly defined AL, SEZ and optic lobes (Bui et al. 2019 and O.R., personal observation). However, larval ALs are smaller and less developed compared with adults, in line with their simplified olfactory systems; larvae have far fewer sensory neurons, for example *Aedes aegypti* larvae have ~ 24 ORNs (McIver 1978) compared with ~ 3800 (Zacharuk et al. 1971) in adults. In *Anopheles gambiae*, larvae have ~ 15 ORNs and adult females have ~ 1500 ORNs (Riabinina et al. 2016). It may be that larvae instead rely on a more heterogeneous selection of IRs and GRs than adults, reflecting their aquatic environment in which gustation as well as olfaction could be utilised in long-range stimulus detection. Bui et al. (2019) hypothesized that there may be particularly strong selective pressure on ORs that are relevant to both larvae and adults. For example, 1-octen-3-ol is a decay volatile, which larvae are innately attracted to as detritivores, as well as a host cue, reliably known to activate ORNs in adult mosquitoes (Bui et al. 2019). In a behavioural assay, decay volatiles including indole, 2-methylphenol, 3-methylphenol, 4-methylphenol and 4-methylcyclohexanol made up 11 of the 33 chemicals from a broad panel of odors that elicited a significant response from larvae (Xia et al. 2008). Interestingly, larval mushroom bodies are anatomically similar to those of adults, potentially highlighting the importance of olfactory learning in larvae (Lutz et al. 2017).

Whilst at present few studies have investigated higher order neurons and brain organisation in mosquitoes, calcium dyes (Lahondère et al. 2019) and recently developed neurogenetic methods to study mosquito brain activity should help to elucidate these topics. Transgenic strains for live calcium imaging have been developed in *Anopheles gambiae* and *Aedes aegypti*, targeting fluorescent calcium indicator GCaMP to all cells (Bui et al. 2019), to all neurons (Jové et al. 2020; Zhao et al. 2020a), or to specific subsets of neurons: ORCO + (Afify et al. 2019), ppk301 (Matthews et al. 2019), IR7a/f and GR4 (Jové et al. 2020). In the adult, live imaging may be conducted in the peripheral (Afify et al. 2019; Jové et al. 2020) or central nervous system (Matthews et al. 2019; Lahondère et al. 2019; Vinauger et al. 2019; Melo et al. 2020). In the larvae, live imaging of brain activity is facilitated by the transparent cuticle of larvae, through which subcutaneous fluorescence can be detected and measured. Additionally, since no surgical cuticle removal is necessary, the individuals may be kept alive sufficiently long to enable repeated testing (Bui et al. 2019). The genetically encoded tools for live imaging are not yet available in other mosquito species, most likely due to the difficulties of generating them in house and the absence of commercial services.

### Species differences and sexual dimorphisms in the antennal lobe and subesophageal zone

Differences in the counted AL glomeruli number have been recorded between *Anopheles gambiae*, *Aedes aegypti* and *Culex quinquefasciatus* (reported in the females for each species to be 67–70, 78–82 and 62 respectively) (Riabinina et al. 2016; Shankar and Mcmeniman 2020; Ye et al. 2020). These figures were obtained using antibody staining with individual glomeruli defined and outlined by hand, a process that inevitably leads to equivocal counts, hence the given ranges. Additionally, tissue deformation during immunostaining procedures may have caused increased individual variability in AL organisation, providing a further source of ambiguity.

The numbers of AL glomeruli in *Culex quinquefasciatus* mosquitoes are strongly sexually dimorphic, with 44 glomeruli in males and 62 glomeruli in females. However, similar glomeruli numbers are seen in males and females of *Anopheles gambiae* (67–68 in males, 67–70 in females) and *Aedes aegypti* (75–79 in males, 78–82 in females). Total AL volume is larger in females: 1.9 times greater in *Anopheles gambiae*, 1.4 times greater in *Aedes aegypti* and 3.1 times greater in *Culex quinquefasciatus*. This is likely because females have a far greater number of ORNs than males, about twice the number in *Anopheles gambiae*, meaning each glomerulus is innervated by more ORNs and is therefore larger. Supporting this, nc82 antibody staining is more intense and clearly segmented in females (Riabinina et al. 2016). These differences reflect the additional olfactory tasks required only of females, particularly relating to oviposition and host-seeking. For example, decay volatiles are not obviously useful to a male mosquito; however, they are specifically important for females in determining an ideal site for oviposition, since the environment’s microbial composition affects offspring fitness (Lutz et al. 2017). Sexual dimorphism between the relative volumes of glomeruli has also been identified for two specific glomeruli, AD1 and VC1, in *Aedes aegypti*, which may similarly be linked to these behaviours (Shankar and Mcmeniman 2020). In contrast to the AL, the SEZ appears to demonstrate little sexual dimorphism; however, *Anopheles gambiae* is the only species for which this has been investigated (Riabinina et al. 2016).

### Multisensory integration and host-seeking

Mosquitoes integrate olfactory signals with other sensory inputs—namely temperature, humidity, mechanosensory and visual information—an ability vital for host-seeking, danger avoidance, mate finding and the location of suitable oviposition sites (Potter 2014; McMeniman et al. 2014; Breugel et al. 2015; Vinauger et al. 2019). Female mosquitoes of many species rely on blood-feeding to gain vital nutrients for egg development, whilst their metabolic demands are primarily satisfied through nectar feeding. Host detection involves sensing and integrating three main cues: CO_2_, host body odour and heat, though moisture detection and visual cues are also involved. CO_2_ detection is accomplished at the receptor level by three GRs (McMeniman et al. 2014; Xu et al. 2020; Kumar et al. 2020; Liu et al. 2020), while the bouquet of human body odor requires information from a range of ORs and IRs. The topic of heat detection and its underlying mechanisms has been the subject of recent research (Corfas and Vosshall 2015; Greppi et al. 2020), with a study using *Anopheles gambiae* finding that the IR21a receptor is highly involved in detecting cooling. With this proposed IR acting as a mediator, the apparent seeking of heat may in fact be an aversion to cool temperatures (Greppi et al. 2020).

In general, at least two of the three primary cues listed above must be simultaneously detected to evoke a host-seeking response, an advantageous principle in complex environments where one cue alone may not be a sufficiently reliable indicator of, in the case of *Aedes aegypti*, a human host (McMeniman et al. 2014). However, at close range fewer cues may be sufficient. CO_2_ is thought to initiate host seeking at the longest range (around 10 m) whilst odor (Bernier et al. 2007; Robinson et al. 2018) and visual (Breugel et al. 2015) cues are involved at a distance of up to 2 m and heat is particularly important at a close range of less than 15 cm (Cardé 2015), at which point the temperature difference between a human arm and ambient temperature falls below the 0.2 °C detection threshold for *Aedes aegypti* (Davis and Sokolove 1975). In such circumstances, heat alone may be a sufficient cue to attract mosquitoes and initiate landing; it was found that female mosquitoes reliably (*p* = 0.024) choose the heat source at 34 °C (host range) over one at 25 °C (ambient) in a Y maze olfactometer with no other host cues present (Zermoglio et al. 2017). Although this experiment placed the heat sources as far away as 37 cm, it is likely that heat does not dissipate as quickly within the confines of a Y maze compared with open air.

Once the host has been located, ORs housed in the stylet may be used to find the best site at which to pierce the skin, in order to precisely locate blood vessels without alerting the host (Won Jung et al. 2015). When AaOr8 and AaOr49 receptors, usually expressed in the stylet of *Aedes aegypti*, were downregulated, the mosquitoes demonstrated significantly higher levels of stylet probing and longer engorgement times, implicating these ORs in blood-seeking processes. The authors also identified two olfactory glomeruli in the AL that are innervated by stylet ORNs, further indicating olfactory involvement (Won Jung et al. 2015). Six stylet IR neurons are also involved in tasting blood via IR7a/f receptors (Jové et al. 2020).

Visual-olfactory integration has an important role in host detection in *Aedes aegypti* (Breugel et al. 2015). Presentation of the attractive cue odor CO_2_ alongside the visual stimulus of horizontal moving bars led to an increase in bar-tracking fidelity compared with a clean air control (Vinauger et al. 2019). In other words, CO_2_ presentation seems to modulate visual responses, making the mosquitoes particularly drawn to visual features. Further experiments looked to identify the neural basis of this phenomenon. Using *Aedes aegypti*, regions of interest (ROIs) were identified in the lobula, a neuropil in the optic lobe, corresponding to areas with high GCaMP6s (a calcium indicator) expression. When mosquitoes were presented with CO_2_ preceding the visual stimulus, fluorescent responses were significantly higher in 14 out of 59 ROIs (though lower in 2), suggesting a positive modulatory effect. However, visual-olfactory modulation is thought to be asymmetric, in that prior presentation of a visual stimulus does not alter olfactory signals (Vinauger et al. 2019).

Distinguishing human odor from that of non-human animals is a crucial ability for many anthropophilic vector species. Recently, a glomerulus activated exclusively by human odours was identified in *Aedes aegypti* (the “human-sensitive” glomerulus). This glomerulus is specifically tuned to detect long-chain aldehydes, a high concentration of which characterises human odor. It is proposed that in conjunction with a broadly tuned “universal” glomerulus, mosquitoes are able to gain information about the relative concentration of long-chain aldehydes and therefore reliably determine whether a given odor is from a human (Zhao et al. 2020b).

## Conclusions and future perspectives

There has been a recent boom in knowledge about mosquito olfactory systems but there is still much to learn. For instance, the OR/IR repertoire of many species is completely unknown. Particularly interesting are species that do not use vertebrates as hosts, such as *Uranotaenia sapphirina,* which feeds on annelid worms (Reeves et al. 2018) and *Malaya* mosquitoes that feed via trophallaxis from ants (Miyagi 1981). It would be interesting to see what chemical cues these species use to find their hosts and whether they have novel receptors to detect these cues.

Another interesting avenue of research is to examine the olfactory repertoires and receptors of larvae with different ecologies. For example, *Anopheles gambiae* or *Aedes aegypti* larvae filter feed on biofilms and bacteria, while larvae of *Toxorhynchites* sp. are predatory and may be attracted by chemical cues produced by their prey larvae. While most species of mosquito develop in freshwater, there are some that develop in water with higher salinity, e.g., *Anopheles aquasalis* (Grillet 2000) and *Aedes togoi* (Trimble and Wellington 1979). Different salinity implies different bacterial communities in each of these settings, which may produce different metabolites, as well as different chemical solubilities and thus may be followed by a co-evolving repertoire of olfactory receptors in the larvae.

Most of the past and recent work has focused on studies of the peripheral olfactory systems of mosquitoes. The next step is to extend the existing methods of recording neuronal responses to specific neuronal subpopulations in higher order brain centres to provide insights into olfactory and multisensory coding.

Future research will require three strands of groundwork. First, we need to learn about mosquito behaviour in the field and their ecologically relevant cues before olfactory or multisensory responses to these cues may be investigated. Second, we will need to develop new behavioural assays that realistically mimic field situations for mosquitoes. Third, molecular and genetic tools will need to be implemented in new species, which in turn will require established lab colonies, commercial services for transgenesis and suitable stock centres. Luckily, with over 3550 extant mosquito species, we are unlikely to run out of exciting new findings any time soon.

## Conflict of interest

The authors declare that they have no conflict of interest.

## Ethical approval

No animals or human subjects were involved in this research and thus no ethical approval and informed consent was necessary.
